# NK cell-associated long non-coding RNAs reveal heterogeneity of colorectal cancer immune microenvironment

**DOI:** 10.3389/fimmu.2025.1615942

**Published:** 2025-11-13

**Authors:** Yuxuan Li, Chuqi Xia, Jinze Li, Jichao Qin, Fan Shi, Wenkai Zhang, Daoming Liang, Yixiong Shu, Qiyu Lu

**Affiliations:** 1 Department of Gastrointestinal Surgery, The Second Affiliated Hospital of Kunming Medical University, Kunming, China; 2 Department of Gastrointestinal Surgery, Tongji Hospital, Tongji Medical College Huazhong University of Science and Technology, Wuhan, China; 3 Hubei University and Physical Education Institute, Wuhan, Hubei, China; 4 Emergency and Critical Care Medicine, Wuhan Third Hospital, Tongren Hospital of Wuhan University, Wuhan, China

**Keywords:** tumor immune single-cell hub 2, colorectal cancer, NK cell-related lncRNAs, tumor immune microenvironment, molecular subtyping

## Abstract

**Introduction:**

Individuals diagnosed with colorectal cancer (CRC) frequently confront a grave prognosis and exhibit poor responses to conventional treatment regimens. Immunotherapy, notably modalities centered on natural killer (NK) cells, represents a burgeoning frontier in the management of CRC. This study developed a validated prognostic model using NK-associated long non-coding RNAs (lncRNAs) to predict CRC outcomes.

**Methods:**

Integrating single-cell RNA-seq (GSE146771_Smartseq2) and TCGA-COAD/READ bulk transcriptomic data, we identified NK-specific genes and correlated lncRNAs. A multi-step analytical approach—including univariate Cox regression for preliminary screening, LASSO regression to minimize overfitting, and multivariate Cox regression for final model optimization—yielded a robust 16-lncRNA prognostic signature with high predictive accuracy.

**Results:**

This model demonstrated robust predictive performance across the training set, validation set, and 76 independent clinical samples. Mechanistic investigations revealed that AC010319.3 is highly expressed in NK cells, where it attenuates NK cell cytotoxicity by suppressing the expression of IFN-γ and granzyme B, thereby promoting the proliferation and invasion of CRC cells.

**Discussion:**

This study systematically delineates the regulatory role of NK-associated lncRNAs within the CRC immune microenvironment, offering novel molecular targets and stratification strategies for CRC immunotherapy.

## 1 Introduction

Globally in 2022, colorectal cancer was responsible for nearly 1.93 million new diagnoses, making it the third most frequent cancer, and for approximately 904,000 fatalities, ranking it as the second leading cause of cancer mortality, only after lung cancer (1). CRC constitutes nearly 10% of both newly diagnosed malignancies and cancer-associated fatalities globally (2). While surgical resection, radiotherapy, and chemotherapy remain therapeutic cornerstones, their clinical utility is constrained by significant limitations. Surgery is exclusively applicable to localized lesions and fails to address micrometastases, whereas conventional chemoradiotherapy indiscriminately inhibits tumor proliferation, often inducing severe off-target complications such as myelosuppression, radiation-induced enteritis, and other related disorders. Furthermore, these traditional modalities demonstrate limited efficacy against microsatellite stable (MSS) CRC (representing 85% of cases) and metastatic disease, with 5-year survival rates remaining below 15%. In contrast, Chimeric Antigen Receptor (CAR) technology, as a core strategy of emerging immunotherapy, achieves precise recognition and killing by expressing CAR structures targeting tumor-specific antigens and utilizing host immune activation, while overcoming immune suppression mediated by the tumor microenvironment (TME) (such as TGF-β and PD-L1 signaling), thereby significantly enhancing cytotoxicity against MSS-CRC (3).As a potent anti-tumor modality, immunotherapy may represent a breakthrough in CRC prognosis optimization and potential cure, positioning itself as an alternative therapeutic strategy for CRC patients.

While T cell-centric immunotherapies, particularly PD-1/PD-L1 inhibitors, have achieved remarkable progress in microsatellite instability-high (MSI-H) CRC, their clinical efficacy remains limited in microsatellite stable (MSS) subtypes that account for 85% of CRC cases (4). This unmet therapeutic challenge has prompted a redirection of scientific focus toward other immune cells, with NK cells—central cytotoxic effectors of the innate immune system—being one of them, demonstrating unique anti-tumor potential in MSS-CRC. Clinical evidence indicates that MSS-CRC patients exhibit suboptimal response rates (<15%) to T cell-based therapies, attributable to low tumor mutational burden and impaired antigen-presenting capacity (5). In stark contrast, NK cells employ the ‘missing-self’ recognition paradigm, enabling MHC-I-independent targeting of MSS tumor cells. As a crucial effector cell of the innate immune system, NK cells possess a unique recognition mechanism distinct from adaptive immune cells (B and T lymphocytes) (6). CAR-T therapy is relatively mature in the treatment of hematological tumors; however, in solid tumors such as CRC, it faces challenges including toxicity, difficulty in tumor microenvironment (TME) penetration, antigen escape, and limited anti-tumor activity. CAR-NK therapy leverages the properties of NK cells, such as the lack of requirement for HLA matching, innate cytotoxicity, and multi-pathway killing mechanisms, thereby reducing the risks of cytokine release syndrome (CRS) and graft-versus-host disease (GVHD), enhancing TME infiltration and persistence, and mitigating antigen escape, thus providing a safer, off-the-shelf therapeutic option for CRC (7, 8).Positioned as an underexplored ‘missing puzzle piece’ in CRC immunotherapy, NK cells may pioneer novel therapeutic avenues (9). With deepening insights into NK cell biology, these innate lymphocytes are poised to become a therapeutic pillar in CRC management.

Long non-coding RNA(lncRNAs) constitute a class of non-coding RNA molecules exceeding 200 nucleotides in length (10). Despite lacking open reading frames (ORFs) and consequent protein-coding capacity, they critically regulate gene expression, orchestrate epigenetic modifications, and dictate cellular fate across physiological and pathological processes. These molecules execute their functions through chromatin remodeling, transcriptional interference, or RNA-protein complex formation, thereby participating in sophisticated regulatory networks governing development, metabolism, immune responses, and tumorigenesis (11). Clinically, immune-related lncRNA-based prognostic models demonstrate robust predictive accuracy in renal cell carcinoma; similarly, in prostate cancer, TYMSOS, as a specific lncRNA associated with immune microenvironment regulation, has been confirmed as a novel biomarker with significant prognostic value (12, 13). Nevertheless, despite growing recognition of lncRNAs’ immunomodulatory roles in oncology, the functional landscape of NK cell-associated lncRNAs in CRC immunotherapy remains unexplored.

In this study, we recognized lncRNAs associated with NK cell function through the TISCH2 and TCGA databases (14). Afterwards, a predictive model for CRC was constructed from a stepwise regression analysis, which incorporated 16 lncRNAs. This model, when combined with age, TNM stage, and risk score, demonstrated superior performance compared with traditional clinical indicators. A risk prognostic model was constructed based on Cox regression, and the model’s validity was verified using the TCGA dataset and 76 CRC samples. To further elucidate the biological mechanisms underlying the model, we focused on AC010319.3. Through *in vitro* and *in vivo* functional experiments, we confirmed that AC010319.3 is specifically highly expressed in NK cells and inhibits the cytotoxic function of NK cells by negatively regulating key cytokines such as IFN-γ and granzyme B, thereby promoting the proliferation and invasion of colorectal cancer cells. This reveals AC010319.3 as a potential therapeutic target for regulating NK cell function, providing new insights for CRC immunotherapy.

## 2 Results

### 2.1 Single-cell transcriptomic Atlas reveals NK cell immune system and differentially regulated gene signatures in colorectal cancer

**Figure 1:**
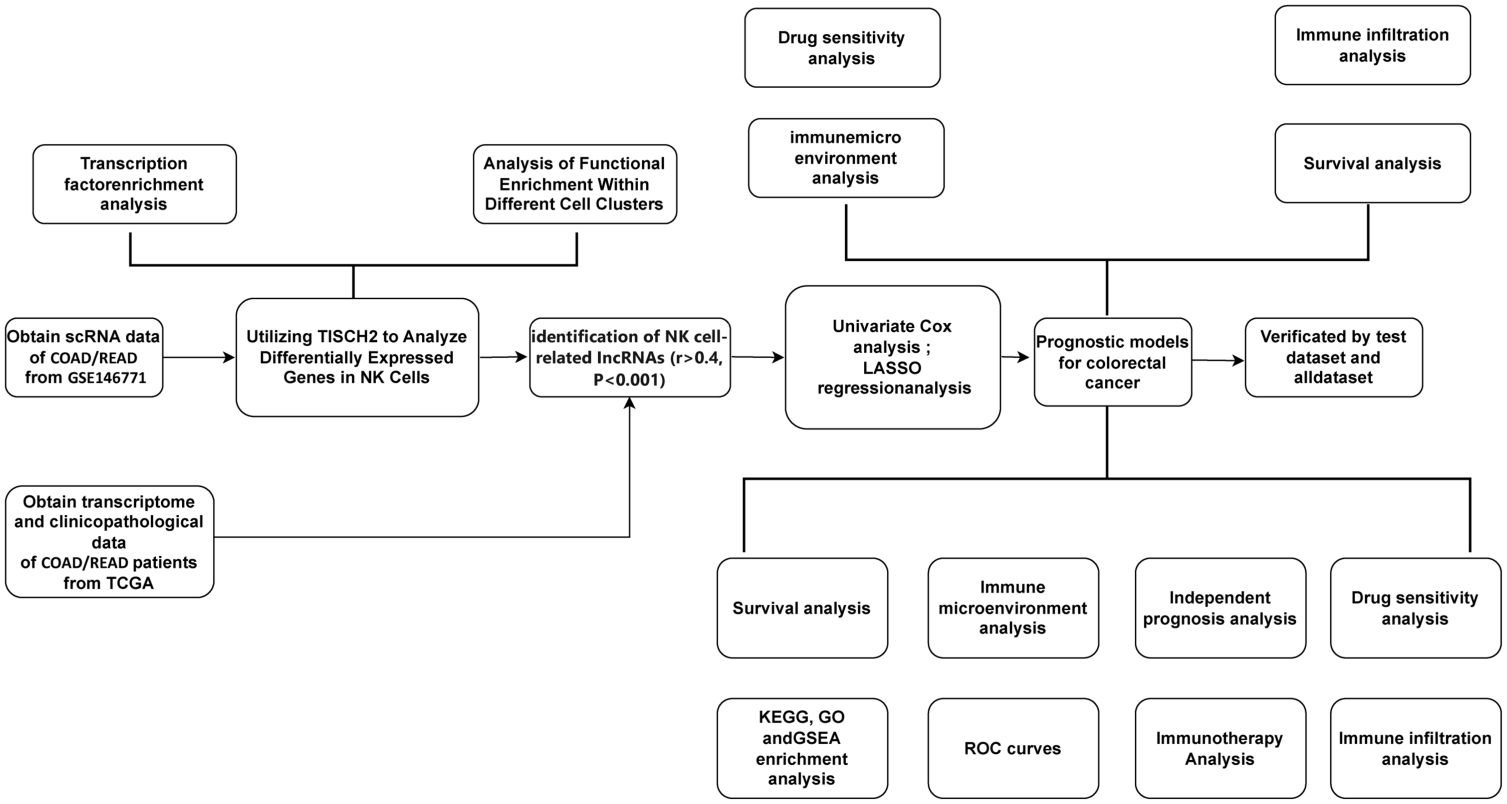
A detailed flowchart illustrates the construction, validation, and molecular subtyping of the NK cell-related lncRNA model in CRC. CRC: Colorectal Cancer; TISCH2: Tumor Immune Single-Cell Hub 2; TCGA: The Cancer Genome Atlas; TF: Transcription factor; LASSO: Least absolute shrinkage and selection operator; NK: Natural Killer cells.

**Figure 2:**
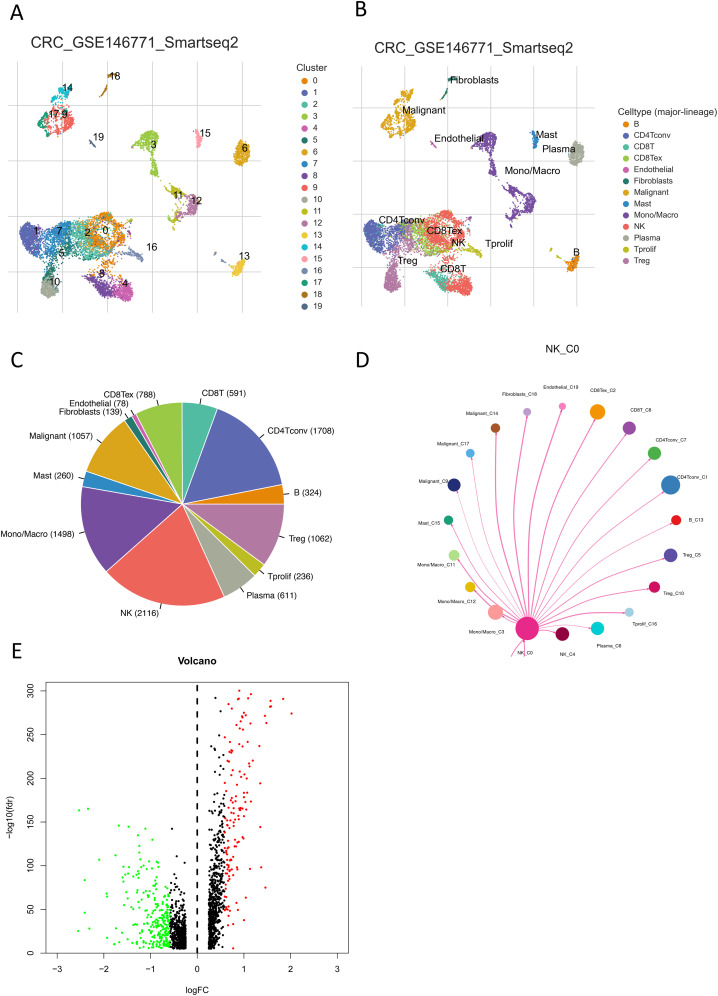
Communication networks of NK cells in CRC. **(A, B)** UMAP plots show the distribution and abundance of different cell subpopulations in CRC. **(C)** A pie chart shows the percentage of NK cells. **(D)** Visualization of interaction probabilities between NK cells and other cells using CellChat. **(E)** A volcano plot shows differentially expressed genes within NK cells. Red indicates fold change > 1.5, FDR < 0.05; green indicates fold change < 1.5, FDR < 0.05.

The progress of the current work is comprehensively depicted as flow diagram (**Figure 1**). Conducted on the TISCH2 platform, dimensionality diminution and clustering analysis of the single-cell RNA sequencing (scRNA-seq) data from the GSE146771_Smartseq2 dataset unveiled a notable enrichment of NK cells in Cluster 0 and Cluster 4 (**Figures 2A, B**). The results showed that NK cells were significantly enriched in CRC patients and formed independent clusters, indicating that NK cells might be the dominant immune cell population in CRC patients and have similar gene expression patterns. A pie chart quantified the abundance and relative frequency of NK cells, demonstrating that NK cells constituted 23.3% of immune cells in CRC patients—significantly higher than in healthy individuals—highlighting their aberrant enrichment and biological relevance in CRC (**Figure 2C**). Cell-cell interactions within the CRC tumor immune microenvironment critically regulate cellular functions, immune states, and disease progression (15). Using the CellChat calculation on the TISCH2 Database, we predicted intercellular communication networks. The analysis identified powerful connection between NK cells and macrophages, CD8+ and CD4+ T cells (**Figure 2D**). To identify NK cell-associated genes (NKGs), we performed Wilcoxon rank-sum tests on the TISCH2 platform (|fold change| > 1.5; FDR < 0.05). we detected 167 elevated and 262 reduced NKGs (**Figure 2E**).

### 2.2 NK cell functional traits in CRC

Leveraging the CellChat technique within TISCH2 system, we quantified intercelllular correlations and identified robust communication among NK cells and CD8+ T cells, fibroblasts, endothelial cells, as well as malignant cells (**Figure 2D**). In CRC, multiple transcription factors (TFs) play pivotal roles. Using the spatial association algorithm on TISCH2, we inferred key TFs regulating gene expression within each cell cluster. Using heat maps, we presented the expression patterns of key transcription factors in all cell clusters within the dataset. (**Supplementary Figure S1A**). Notably, in the NK_C4 and NK_C0 subgroups, KDM4c and ZNF274, respectively, emerged as the most significantly enriched TFs, indicating their core roles in this regulatory network (**Supplementary Figure S2A, B**). These TFs may modulate NK cell functionality and thereby influence the CRC TME, offering novel perspectives for investigating CRC immunology. We leveraged the advanced enrichment capabilities of the TISCH2 platform to further investigate NK cell mechanisms in CRC. Through KEGG pathway analysis, we identified significant upregulation of natural killer cell-mediated cytotoxicity, ribosome, spliceosome, and neuroactive ligand-receptor interactions, alongside downregulation of hematopoietic cell lineage, primary immunodeficiency, and intestinal immune network for IgA production (**Supplementary Figures S3, S4**). In Gene Ontology (GO) analysis, we observed upregulated nuclear-transcribed mRNA catabolic processes in biological processes, proteasome core complexes and T-cell receptor complexes in cellular components, and antigen binding in molecular functions. Conversely, JNK kinase activity (biological processes), endoplasmic reticulum, phagocytic vesicle membranes, and secretory granule membranes (cellular components), as well as cytokine receptor activity and endopeptidase activity (molecular functions), were downregulated (**Supplementary Figures S5–S7**). We observed correlations between NK cells and multiple T cell-related immunogenomes (**Supplementary Figure S8**).

### 2.3 Construction and multi-center validation of a prognostic model based on NK cell signature genes

**Figure 3:**
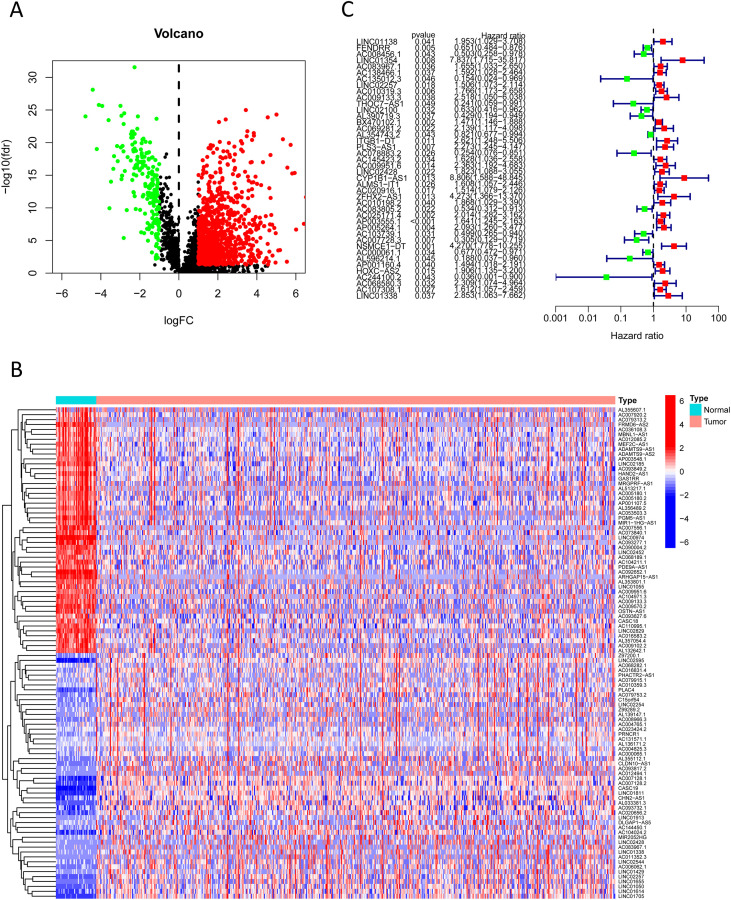
Prognosis-associated NK cell-related lncRNAs. **(A)** A volcano plot shows 1,133 differentially expressed NK cell-related lncRNAs identified in CRC (red: logFC > 0.585, FDR-adjusted p < 0.05; green: logFC < 0.585, FDR-adjusted p < 0.05). **(B)** A heatmap visually displays the top 50 most significantly differentially expressed NK cell-related lncRNAs. **(C)** A forest plot shows the results of univariate Cox regression analysis, identifying 42 lncRNAs associated with CRC prognosis (green indicates hazard ratio < 1; red indicates hazard ratio > 1).

**Figure 4:**
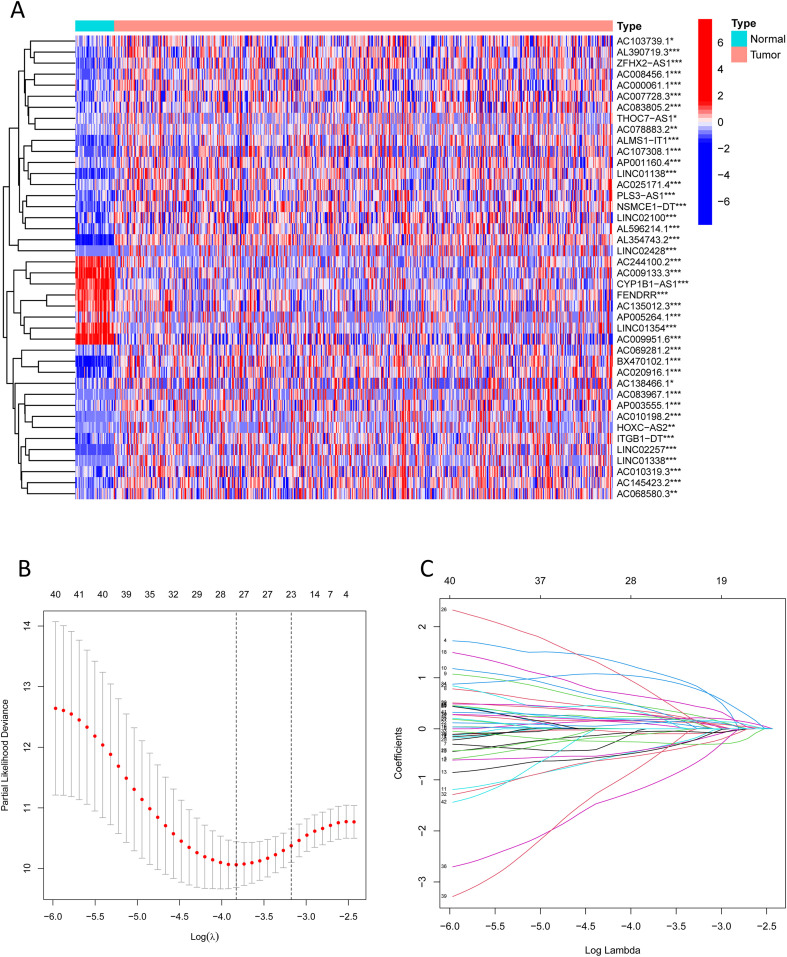
Prognosis-associated NK cell-related lncRNAs. **(A)** A heatmap shows the expression differences of identified lncRNAs between CRC and normal samples (*** indicates p < 0.001, ** indicates p < 0.01, * indicates p < 0.05). **(B, C)** Lasso regression analysis reveals overfitting phenomena in the model under different gene number settings and compares the severity of overfitting under these settings.

**Figure 5:**
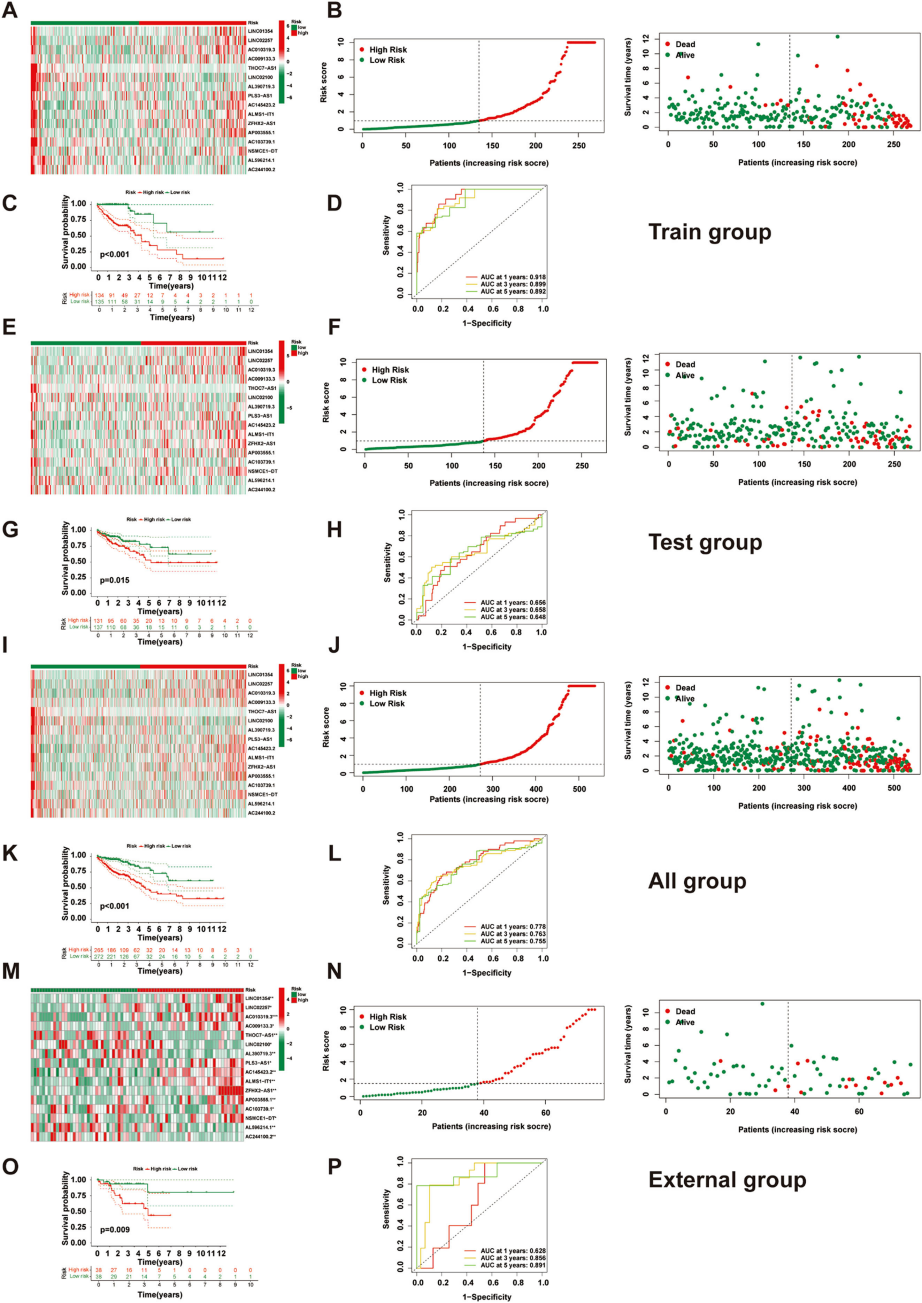
Construction and validation of the NK cell-related prognostic model. **(A)** A heatmap shows the expression of 16 lncRNAs in high- and low-risk groups in the training set. **(B)** Distribution and survival status of CRC patients in the training set. **(C)** Kaplan-Meier survival curve comparison between high- and low-risk groups in the training set. **(D)** ROC curve evaluation in the training set. **(E)** A heatmap shows the expression of 16 lncRNAs in high- and low-risk groups in the test set. **(F)** Distribution and survival status of CRC patients in the test set. **(G)** Kaplan-Meier survival curve comparison between high- and low-risk groups in the test set. **(H)** ROC curve evaluation in the test set. **(I)** A heatmap shows the expression of 16 lncRNAs in high- and low-risk groups in all patients. **(J)** Distribution and survival status of CRC patients in all patients. **(K)** Kaplan-Meier survival curve comparison between high- and low-risk groups in all patients. **(L)** ROC curve evaluation in all patients. **(M)** Heatmap displaying the expression of 16 lncRNAs in high- and low-risk groups within the external validation set(n=76). **(N)** Distribution and survival status of CRC patients in the external validation set(n=76). **(O)** Comparison of Kaplan-Meier survival curves between high- and low-risk groups in the external validation set set(n=76). **(P)** ROC curve evaluation in the external validation set(n=76).

We extracted expression profiles of previously discovered NKGs from TCGA colorectal adenocarcinoma read cohort (16). Utilizing Pearson correlation assay (correlation coefficient exceeding 0.4, p-value below 0.001), we detected NK cell-associated lncRNAs. Subsequent differential expression analysis revealed 1,133 dissimilarly expressed NK cell- attached lncRNAs within CRC (**Figure 3A**). We utilized heatmap visually displays the top 50 most differentially expressed genes in CRC cases (**Figure 3B**). Univariate Cox regression analysis of the training cohort identified 42 prognosis-associated lncRNAs (**Figure 3C**). A heatmap illustrates their expression differences between CRC tumors and normal tissues (**Figure 4A**). To preclude overfitting, LASSO regression analysis was implemented (**Figures 4B, C**). Multivariable Cox regression further refined the model to 16 independent NK cell-related lncRNAs with prognostic significance. Risk Score = 1.66835172119723 × LINC01354 expression + 0.382958718659199 × LINC02257 expression + 1.05788009854083 × AC010319.3 expression + 0.954271934790829 × AC009133.3 expression − 2.0577555829965 × THOC7-AS1 expression − 0.915211633569471 × LINC02100 expression − 1.06232138758457 × AL390719.3 expression + 0.876450507692965 × PLS3-AS1 expression + 0.66491751956799 × AC145423.2 expression + 0.689453742016689 × ALMS1-IT1 expression + 3.25571273528702 × ZFHX2-AS1 expression + 0.489382404521168 × AP003555.1 expression − 1.04327479032394 × AC103739.1 expression + 1.06508271262886 × NSMCE1-DT expression − 2.316681672941 × AL596214.1 expression − 3.18945858556773 × AC244100.2 expression. The training cohorts were divided into low - high risk (1:1 ratio). The expression analysis of 16 lncRNAs demonstrated distinct subgroup-specific patterns: ten lncRNAs (including LINC01354, LINC02257, AC010319.3, AC009133.3, PLS3-AS1, AC145423.2, ALMS1-IT1, ZFHX2-AS1, AP003555.1, and NSMCE1-DT) were significantly upregulated in high-risk patients, whereas six lncRNAs (such as THOC7-AS1, LINC02100, AL390719.3, AC103739.1, AL596214.1, and AC244100.2) exhibited elevated expression in low-risk counterparts (**Figure 5A**). In addition to illustrating the distribution and survival outcomes of CRC patients in the training cohort (**Figure 5B**), the study revealed a significant correlation between high-risk scores and elevated mortality rates (**Figure 5C**). The model demonstrated strong predictive functioning, with 1,3 and 5-year AUC values of 0.918, 0.899, and 0.892, respectively, in the training set (**Figure 5D**). Internal verification using the test group and the entire group (**Figures 5E–L**) consistently replicated findings in lncRNA expression patterns, survival analysis, and different expression, confirming the soundness of the prognostic model. To verify the validity of the model, we obtained lncRNA expression profiles and survival data from 76 CRC clinical samples from the sample bank of Tongji Hospital Affiliated to Tongji Medical College of Huazhong University of Science and Technology, to further assess the robustness of the risk scoring model. The analysis results showed that aspects such as the expression patterns of the 16 lncRNAs (**Figure 5M**), the distribution of CRC patients in risk stratification groups and their survival status (**Figure 5N**), the significant correlation between high-risk scores and higher mortality (**Figure 5O**), and the area under the ROC curve (**Figure 5P**) were similar to those of the TCGA training set and internal validation set, thereby further confirming the reliability of this risk scoring model.

### 2.4 Clinical application value exploration of the NK cell-related lncRNA signature

**Figure 6:**
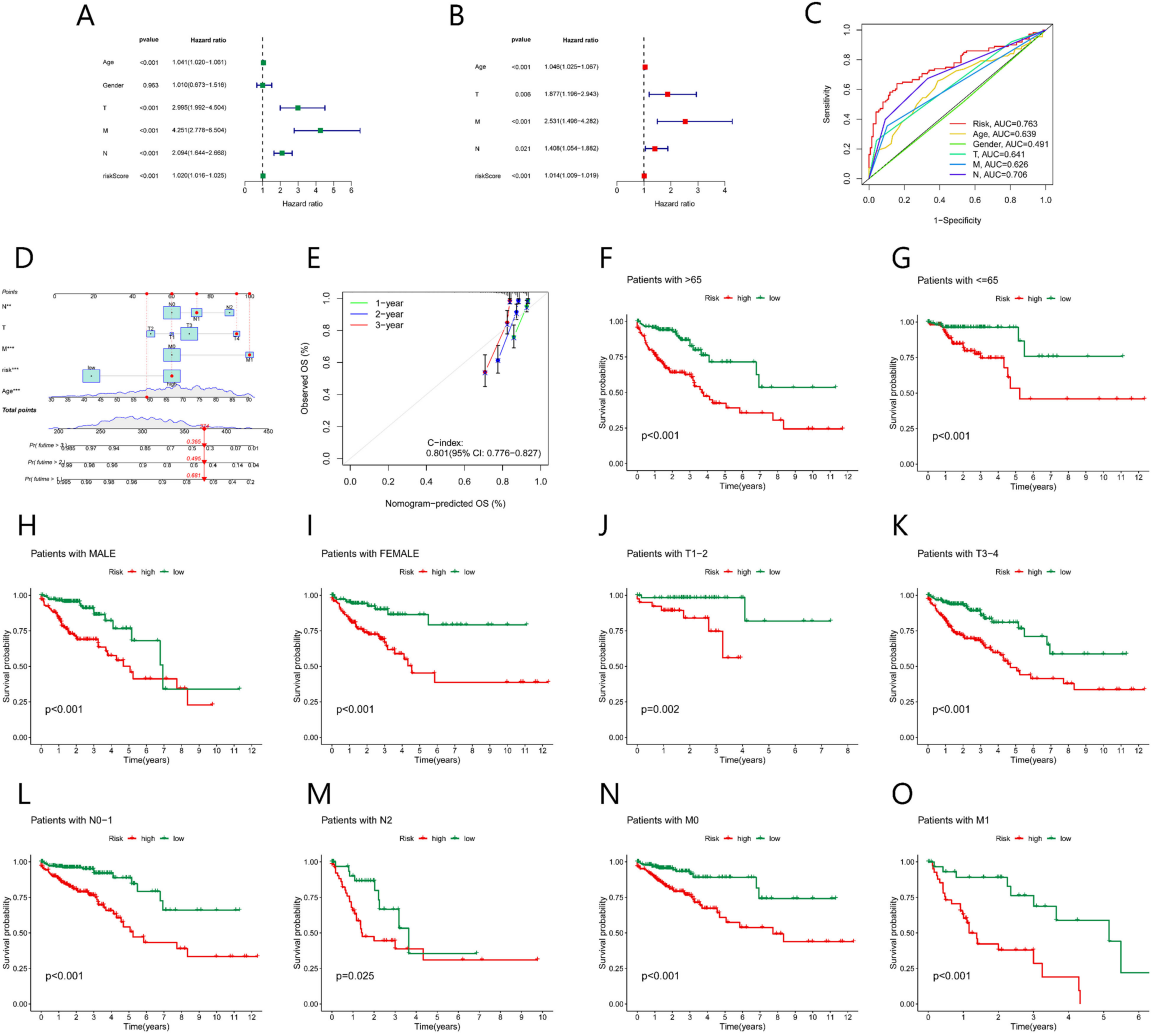
Association between the prognostic model and clinical factors. **(A)** Univariate Cox regression shows factors affecting CRC prognosis. **(B)** Multivariate Cox regression shows independent factors affecting CRC prognosis. **(C)** ROC curve analysis evaluates the accuracy of various clinical variables and risk scores in predicting CRC prognosis. **(D)** Nomogram predicts CRC prognosis. **(E)** Calibration curve assesses the predictive ability of the nomogram. **(F-O)** Kaplan-Meier survival curves for various clinical subgroups based on risk scores.

We evaluated the clinical utility of the NK cell-related lncRNA signature by incorporating the score of risk as a prognostic parameter alongside other clinical parameters in univariate COX regression analysis (**Figure 6A**). Age, TMN stage, and risk score were recognized as influential prognostic parameters (HR> 1, *p* < 0.05). Subsequent multivariable COX regression analysis confirmed age, TNM stage, and risk score as independent prognostic indicators (**Figure 6B**). ROC analysis demonstrated that the risk score outperformed all else clinical parameters in predictive accuracy (**Figure 6C**). A nomogram uniting age, TNM stage, and risk score used to enhance clinical risk stratification (**Figure 6D**). The calibration curves for 1-, 2-, and 3-year survival closely aligned with ideal predictions, and a concordance index (C-index) of 0.801 further validated the nomogram’s reliability (**Figure 6E**). Stratified analyses across sex, TNM stage, and age subgroups (≤65 *vs*. >65 years) consistently affirmed the robust predictive power of the risk score (**Figures 6F–Q**).

### 2.5 Integrated multi-omics enrichment analysis of dissimilarly expressed genes reveals immune microenvironment dysregulation in high-risk subgroups

**Figure 7:**
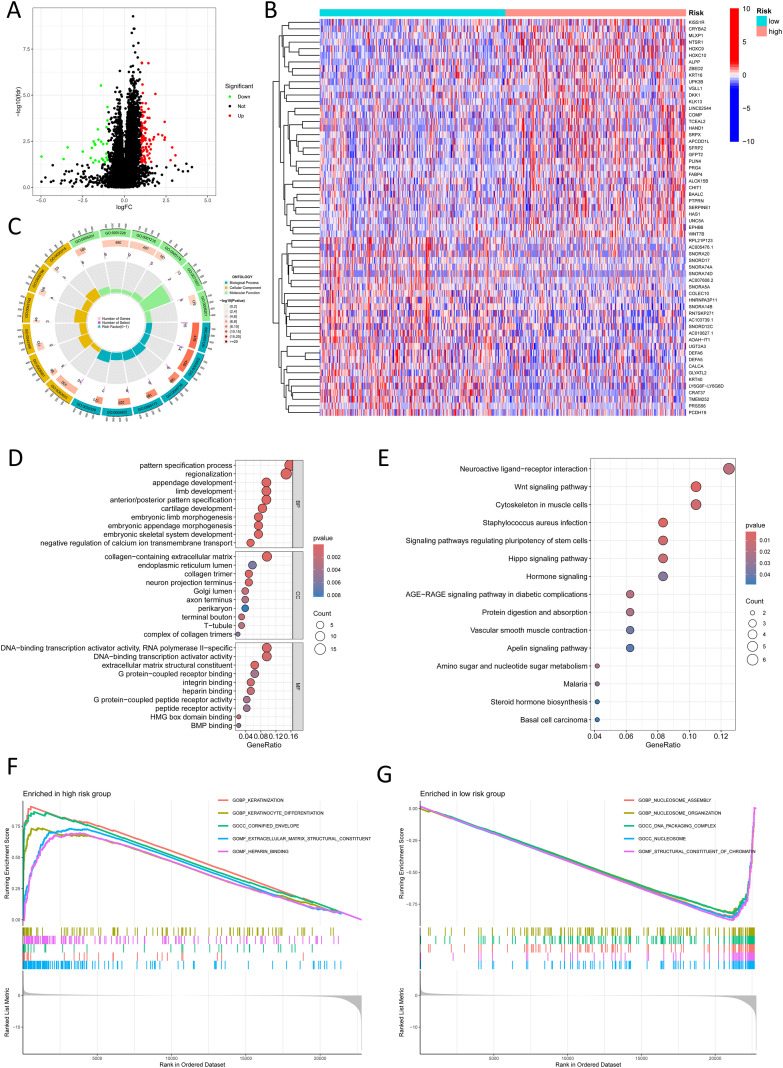
Functional enrichment analysis in different risk groups. **(A)** A volcano plot shows differentially expressed genes between risk groups (red: logFC > 1, FDR-adjusted p < 0.05; green: logFC < 1, FDR-adjusted p < 0.05). **(B)** A heatmap shows the distribution of differentially expressed genes in different risk groups. **(C)** A Circos plot reveals changes in differentially expressed genes in the GO pathways. **(D)** Bubble plot reveals GO pathways enriched by significantly differentially expressed genes. **(E)** Bubble plot shows KEGG pathways enriched by significantly differentially expressed genes. **(F)** GSEA shows upregulated pathways in the high-risk group. **(G)** GSEA shows downregulated pathways in the low-risk group.

To investigate the mechanisms underlying poor prognosis in high-risk patients, we identified 124 dissimilarly expressed genes (DEGs) (|log2FC| > 1, FDR-adjusted *p* < 0.05) between high and low risk groups (**Figure 7A**). A heatmap highlights the top 30 most important DEGs (**Figure 7B**). GO enrichment assay unveiled that the high-risk crowd exhibited marked alterations in endoplasmic reticulum lumen and RNA polymerase II-specific DNA-binding transcription activator activity (**Figures 7C, D**). KEGG pathway assay demonstrated enrichment of cell signaling pathways in the high-risk group, including neuroactive ligand-receptor interactions, Wnt signaling, pluripotency regulation, Hippo signaling, AGE-RAGE signaling, and apelin signaling (**Figure 7E**). Gene Set Enrichment Analysis (GSEA) further uncovered important beneficiation of keratinization and extracellular matrix (ECM) structural organization in the high-risk group, suggesting epithelial-mesenchymal dysregulation and invasive tumor pathology. In contrast, the low-risk group showed prominent enrichment of nucleosome assembly and chromatin structural components, potentially linked to enhanced genomic stability and stringent transcriptional control (**Figures 7F, G**).

### 2.6 Risk stratification of colorectal cancer by NK cell-related lncRNA model reveals immune microenvironment regulation mechanisms and new strategies for targeted therapy

**Figure 8:**
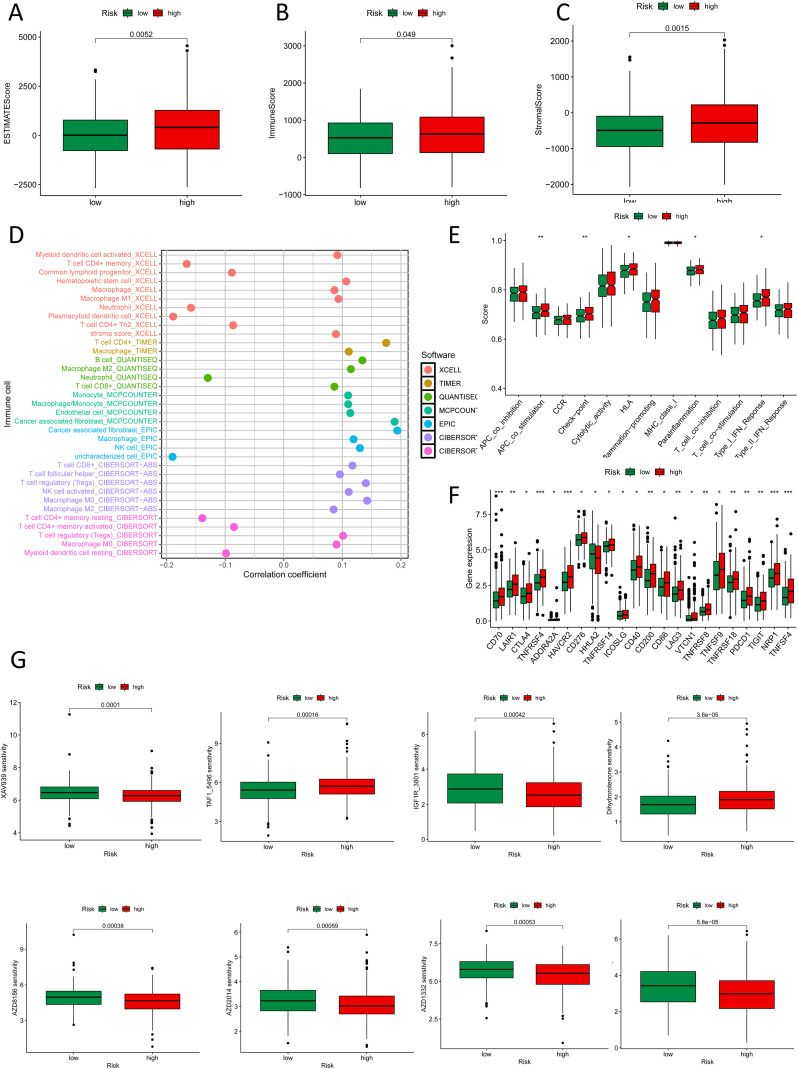
Immune and drug sensitivity analysis. **(A-C)** ESTIMATE scores, immune scores, and stromal scores in high- and low-risk groups. **(D)** Quantitative analysis of immune infiltration in the new CRC subtyping using various algorithms. **(E)** Box plots show immune function status in high- and low-risk groups. **(F)** Box plots show immune checkpoint status in high- and low-risk groups. **(G)** Box plots show drug sensitivity status in high- and low-risk groups. *p < 0.05, **p < 0.01, ***p < 0.001.

In the analysis of the immune microenvironment, CRC cases in the high-risk crowd, as determined by the NK cell-related lncRNA signature, exhibited major discrepancies in stromal score, immune score, and estimate score (**Figures 8A-C**). The combined evaluation of these scores can systematically reveal the variation of TME in CRC and holds important prognostic value for patients. Our study investigated the correlation among the NK cell-related lncRNA signature score and immune percolation within CRC. Using numerous software tools to judge the immune cell infiltration degree in CRC crowds, we observed that the infiltration level of NK cells in TME of CRC crowds was significantly positively connected with the risk score formulated on the NK cell-related lncRNA model (**Figure 8D**). An elevated level of NK cell infiltration is typically associated with enhanced tumor cell killing efficiency, representing a proactive engagement of the immune system to curb tumor progression. Further analysis revealed that patients in the high-risk group displayed enhanced antigen-presenting co-stimulation, significant upregulation of immune checkpoint transcription levels, activation of the HLA pathway, as well as abnormal activation of the parainflammatory response and type I interferon (IFN) signaling pathway (**Figure 8E**). These phenomena suggest a synergistic interplay between immune activation signals and inhibitory regulatory networks within TME: on one hand, overexpression of immune checkpoints suppresses the anti-tumor functions of effector cells; on the other hand, chronic inflammatory responses and activation of the type I IFN pathway may further exacerbate immune evasion through immune exhaustion mediated by pro-inflammatory cytokines or the recruitment of immunosuppressive cells. Thus, the immune profile of the high-risk sort reflects the complexity of tumor immune evasion through multiple regulatory mechanisms. Immune checkpoint analysis showed that, except for HHLA2, which was significantly overexpressed in the low-risk group, the remaining 21 immune checkpoint genes (ICGs) were significantly upregulated in the high-risk population, indicating a more severe state of tumor immunosuppression. Since these genes serve as the targets of immune checkpoint inhibitors (ICIs), the high-risk population may exhibit greater sensitivity to ICI therapy (**Figure 8F**). We conducted a drug sensitivity assessment, which revealed that the high-risk crowd exhibited higher sensitivity to drugs including AZ960, AZD1332, AZD2014, AZD8186, IGF1R_3801, Luminespib, and XAV939, indicating that these patients may benefit more from these therapies, while Dihydrorotenone and TAF1_5496 were proved to be further suitable for patients in the low-risk group (**Figure 8J**).

### 2.7 Characterization of CRC molecular subtypes based on NK cell-related lncRNA

**Figure 9:**
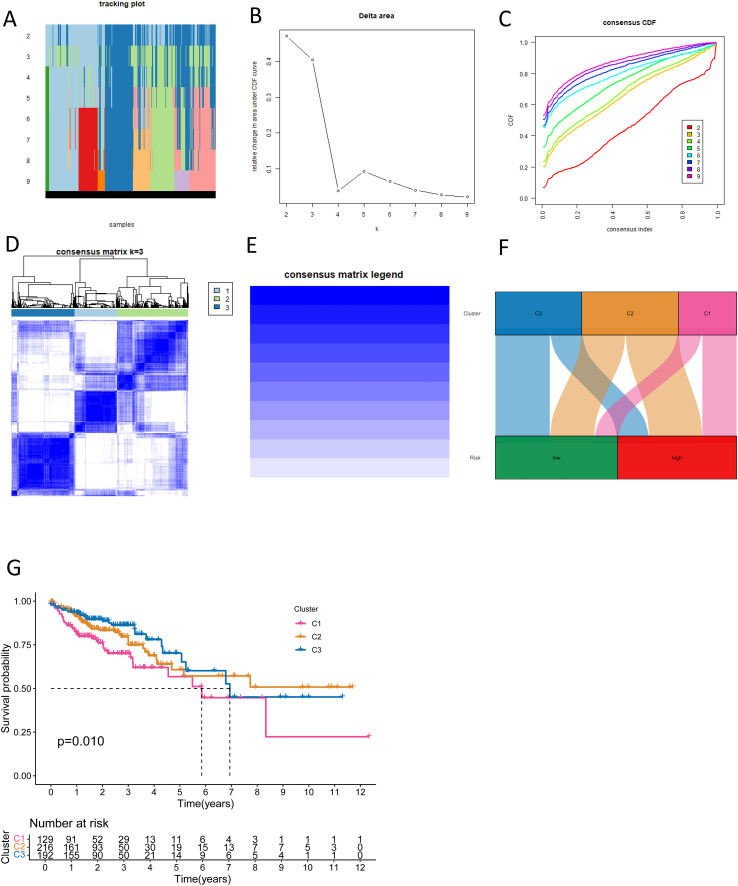
New CRC subtyping based on NK cell-related lncRNAs. **(A)** Sample distribution of different subtyping numbers. **(B)** CDF curves for different subtyping numbers. **(C)** Consensus CDF for different subtyping numbers. **(D, E)** Consensus matrices for three subtypes. **(F)** A Sankey diagram shows the relationship between different CRC subtypes and risk scores. **(G)** Survival curves for different CRC subtypes.

In this way, we classified CRC tumor samples using the NK cell-related lncRNA model. Among the tested values, κ = 3 exhibited a flatter and closer-to-maximum CDF distribution (**Figures 9A-C**). Therefore, we set κ = 3 and divided the CRC tumor samples into three subtypes: Cluster 1-3 (C1, C2, C3) (**Figures 9D, E**). The relationship between high and low-risk score groups and tumor subtypes was visualized using a Sankey diagram. C1 and C2 were predominantly found in the high-risk group, while C3 was mainly distributed in the low-risk group (**Figure 9F**). Survival examination showed that subtype C1 had the worst prognosis (**Figure 9G**). These results demonstrate that the NK cell-related lncRNA model is capable of classifying CRC patient samples into molecular subtypes.

### 2.8 Molecular subtyping of colorectal cancer formulated on NK cell-related lncRNA reveals heterogeneity in immunotherapy and new strategies for personalized treatment

**Figure 10:**
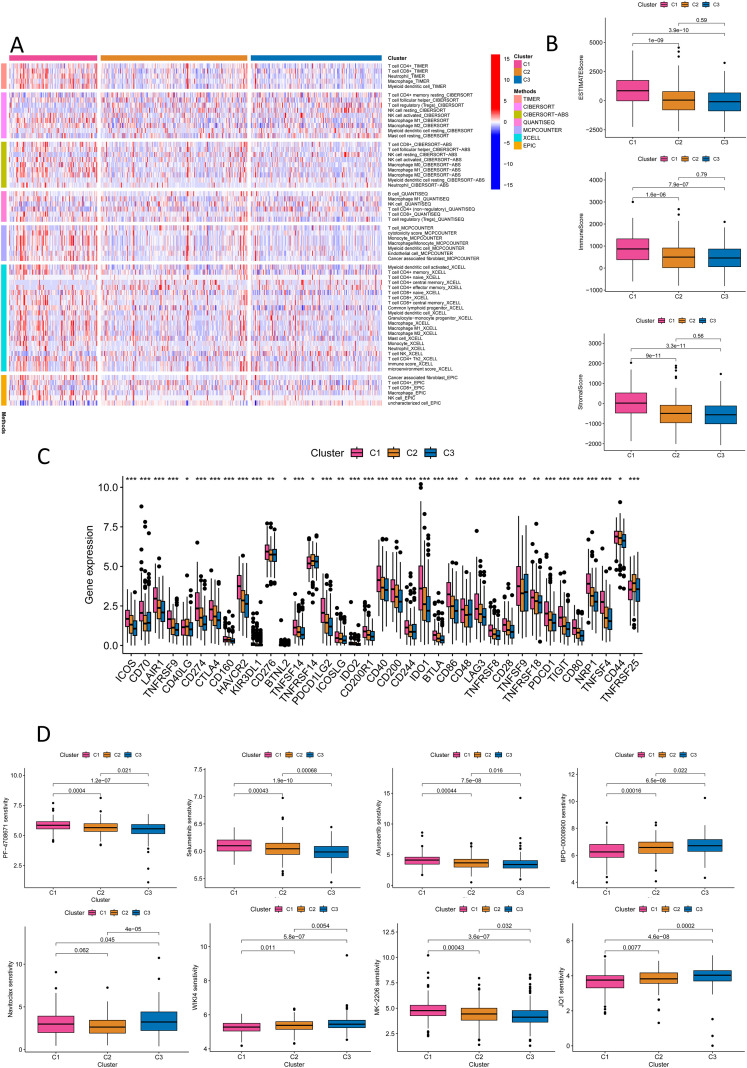
Immune and drug sensitivity analysis of the new CRC molecular subtyping. **(A)** Quantitative analysis of immune infiltration in the new CRC molecular subtyping using various algorithms. **(B)** ESTIMATE Score, ImmuneScore, and StromalScore for different CRC subtypes. **(C)** Immune checkpoint analysis for different CRC subtypes. **(D)** Drug sensitivity analysis for different CRC subtypes. *P < 0.05, **P < 0.01, ***P < 0.001.

To evaluate the potential of molecular subtyping based on NK cell-related lncRNA in immunotherapy for CRC subtypes, we conducted an in-depth analysis of the tumor immune microenvironment. Using various algorithms, we observed the most plentiful immune cell infiltration in subtype C1 CRC, while subtypes C2 and C3 exhibited weaker immune cell infiltration (**Figure 10A**). We further analyzed the StromalScore, ImmuneScore, and ESTIMATEScore for different subtypes (**Figure 10B**). The scores for subtype C1 were uniformly high, the estimates for subtype C3 were low, and subtype C2 was in the middle. This further revealed the heterogeneity of the CRC tumor microenvironment: the high-infiltration and functional inhibition features of subtype C1, the partial activation state of subtype C2, and the immune-cold phenotype of subtype C3, each corresponding to different therapeutic targets and clinical strategies. Additionally, we performed immune checkpoint analysis (**Figure 10C**). For CRC subtypes with high levels of expression of immune checkpoint genes, the use of immune checkpoint inhibitors targeting the corresponding genes would be more appropriate. It was found that, except for CD40LG, TNFRSF14, and TNFRSF25, which were extremely expressed in subtype C2, the remaining immune checkpoint genes were highly expressed in subtype C1. Subtype C3 had the lowest expression in most immune checkpoints, indicating less immunosuppression and a better prognosis. Our analysis of drug sensitivity based on the three molecular subtypes showed that patients in subtype C1 had the highest sensitivity to BPD-00008900, JQ1, and WIKI4; those in subtype C2 had the highest sensitivity to Navitoclax; and those in subtype C3 had the highest sensitivity to Afuresertib, MK-2206, PF-4708671, and Selumetinib (**Figure 10D**, **Supplementary Figure S9**). Molecular subtypes based on NK cell-related lncRNAs aid in assessing the immune microenvironment and immunotherapy, offering fresh thinking for precise CRC treatment, with in-depth analysis of each subtype enhancing personalized treatment strategies and improving therapeutic outcomes.

### 2.9 AC010319.3 inhibits NK cell function to promote CRC progression

**Figure 11:**
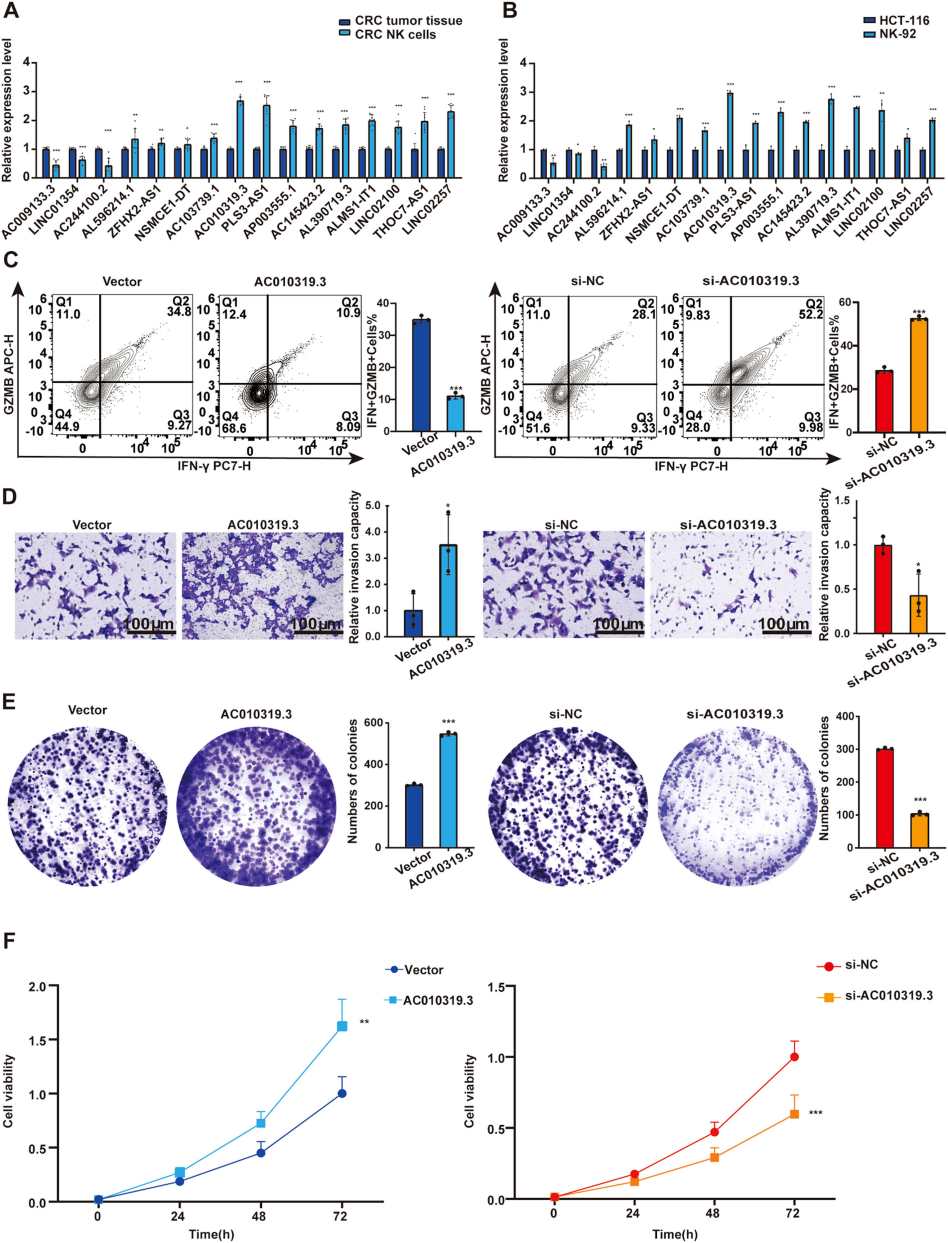
AC010319.3 Promotes Colorectal Cancer Progression by Suppressing NK Cell-Related Functions. **(A)** Relative expression of lncRNA in NK cells within tumor tissues. **(B)** Comparison of lncRNA expression between NK cell lines and colorectal cancer cell lines. **(C)** Detection of intrinsic functions of NK cells through overexpression and knockdown of AC010319.3. **(D)** Transwell assay to validate invasive ability after overexpression and knockdown of AC010319.3. **(E)** Colony formation assay to validate proliferative ability after overexpression and knockdown of AC010319.3. **(F)** CCK-8 assay to validate proliferative ability after overexpression and knockdown of AC010319.3. *P < 0.05, **P < 0.01, ***P < 0.001.

To verify the regulatory role of lncRNA on NK cells, we employed flow cytometry to sort tumor tissues from CRC patients (**Supplementary Figure S10A**). Through qPCR detection of lncRNA expression levels in NK cells and CRC tumor tissues, we found that AC010319.3 exhibited the most significant upregulation relative to tumor tissues (**Figure 11A**). Subsequently, qPCR was used to detect lncRNA expression in the NK cell line NK92 and CRC cell lines (HCT-116, HT-29, and SW-480), revealing that AC010319.3 expression was higher in NK92 cells than in the three CRC cell lines (**Figure 11B**, **Supplementary Figure S10B**). Therefore, this study focused on the function of AC010319.3 in NK cells and its mechanism by which it regulates NK cell killing capacity to influence CRC progression. TCGA database analysis revealed that high expression of AC010319.3 was significantly associated with poor patient prognosis (**Supplementary Figure S10C**), and our 76 independent clinical samples also showed that high expression of AC010319.3 was significantly associated with poor patient prognosis (**Supplementary Figure S10D**). To clarify the function of AC010319.3 in NK cells, we successfully constructed NK92 cell models with overexpression and knockdown of AC010319.3 (**Supplementary Figures S10E, F**). Flow cytometry detection showed that, compared to the Vector group, overexpression of AC010319.3 significantly reduced the expression of IFN-γ and GZMB in NK92 cells; whereas, compared to the si-NC group, knockdown of AC010319.3 markedly increased the expression of IFN-γ and GZMB in NK92 cells, indicating that AC010319.3 can effectively inhibit the expression of key effector molecules in NK92 cells (**Figure 11C**). Subsequently, NK92 cells with overexpression and knockdown of AC010319.3 were co-cultured with HCT116 cells, respectively. Transwell assays showed that the invasion ability of HCT116 cells was enhanced in the AC010319.3 overexpression group, while it was weakened in the knockdown group (**Figure 11D**). CCK-8 and plate colony formation assays showed that the proliferation vitality of HCT116 cells was significantly enhanced in the AC010319.3 overexpression group, while it was significantly inhibited in the knockdown group (**Figures 11E, F**). These results consistently indicate that AC010319.3 promotes CRC progression by inhibiting NK cell function, thereby attenuating their suppressive effect on colorectal cancer cells.

## 3 Discussion

In this study, we constructed and validated a CRC prognostic signature based on 16 lncRNAs. Analysis of the TCGA database and 76 independent clinical samples demonstrated that the model exhibited robust stability and predictive performance in terms of expression patterns, risk stratification, survival distribution, and ROC curves, further supporting its clinical application potential. To investigate the impact of lncRNAs on NK cell function, we selected AC010319.3—the lncRNA with the most significant upregulation in NK cells from colorectal cancer tissues—for subsequent validation. We found that it was highly expressed in tumor-infiltrating NK cells and the NK92 cell line, and it was significantly associated with poor patient prognosis. Functional experiments revealed that AC010319.3 inhibits the expression of IFN-γ and GZMB in NK cells, thereby promoting the proliferation and invasion of CRC cells. This discovery unveils a novel mechanism by which AC010319.3 promotes CRC progression through suppression of NK cell function, providing a potential molecular target for CRC therapeutic strategies targeting NK cell function.

Using the risk score, we isolated patients into high- and low-risk crowds and found significant differences in pathway enrichment between the two groups. KEGG enrichment analysis showed that the cell signaling pathways enriched in the high-risk group included neural active ligand-receptor interaction, Wnt signaling, stem cell pluripotency regulation, Hippo signaling, AGE-RAGE signaling and Apelin signaling pathways. Studies have shown that Wnt signaling and Hippo signaling can affect the development of CRC, suggesting that the synergistic dysregulation of Wnt and Hippo signaling pathways in CRC patients may drive the enormous movement of tumors through the formation of YAP/β-catenin complex (17, 18). NK cell-related lncRNA may promote proliferation by regulating β-catenin target genes, and induce YAP/TAZ nuclear translocation by inhibiting Hippo pathway to enhance the characteristics of cancer stem cells (19). In addition, GSEA showed that keratinization and extracellular matrix structural mechanisms were considerably enriched in the high-risk group, possibly reflecting epithelial-mesenchymal interactions and the pathological mechanisms of aggressive tumors. In contrast, the low-risk group showed more pronounced enrichment of nucleosome assembly and chromatin structural components, which may be related to enhanced genomic stability, tight transcriptional regulation, and precise cell cycle control. These findings indicate that NK cell-associated lncRNAs may influence the malignant progression of CRC by regulating key signaling networks and molecular mechanisms.

Our study elucidates the dynamic interplay mechanisms between NK cell-related lncRNAs and TME in CRC. While the high-risk group exhibits increased NK cell infiltration and enhanced immune co-stimulation, the abnormal activation of immune checkpoints and chronic inflammatory responses mediated by type I interferon collectively shape the dynamic imbalance of the microenvironment. This finding offers an academic basis for precision treatment strategies targeting immune checkpoints in combination with inflammatory pathway regulation. Currently, researchers are enhancing the talent of NK cells to discern and kill tumors through genetic modification, ex vivo expansion, and combined drug stimulation. Immune checkpoint analysis revealed that, except for HHLA2, which was significantly overexpressed in the low-risk group, the remaining 21 ICGs were significantly upregulated in the high-risk group. This further highlights the complexity of tumor immune evasion through multiple immunosuppressive mechanisms. CTLA-4 has a chief part in regulating immune responses and inducing self-tolerance (20). HHLA2 is a B7 family checkpoint molecule with unique dual immune regulatory functions, exhibiting a negative correlation with PD-L1 expression (21). Targeting its inhibitory signaling pathway or developing bispecific antibodies that simultaneously block PD-L1 and activate HHLA2 may be applicable to tumors that do not respond well to existing checkpoint inhibitors. Else ICGs molecules also hold potential value in the progress of immunotherapeutic drugs for colorectal cancer, which requires further validation through multicenter, large-sample clinical studies.

The prognostic model for CRC developed in this study has revealed distinct treatment sensitivities among different patient cohorts through the molecular stratification into subtypes C1-C3. Specifically, the C1 subtype exhibits the highest sensitivity to BPD-00008900, an experimental small-molecule inhibitor targeting the DNA damage repair pathway, as well as to JQ1 (a BET inhibitor) and WIKI4 (a Wnt pathway inhibitor). In contrast, the C2 subtype demonstrates sensitivity to Navitoclax, a BCL-2/BCL-xL inhibitor. The C3 subtype shows significant responses to Afuresertib and MK-2206, both AKT inhibitors, and to Selumetinib, a MEK inhibitor. The study findings indicate that AKT inhibitors such as MK-2206, MEK inhibitors like Selumetinib, and BET inhibitors including JQ1 all exert significant effects on CRC treatment (22–24). It is important to note that drugs such as BPD-00008900 and Navitoclax are currently in preclinical research stages, and their safety and efficacy require validation through multicenter trials.

This study has certain limitations that warrant further validation in future research. Although we have verified the model’s performance using 76 clinical samples, its generalizability still requires further confirmation through future multi-center, large-sample prospective studies. Additionally, we fully recognize the importance of employing a longitudinal sampling design to further validate the prognostic value of the aforementioned lncRNAs. As such validation requires prospective study design, dynamic sample collection at multiple time points, and control for treatment-related confounding factors, it has not been included in the current study. We plan to specifically design longitudinal cohort studies in follow-up work to further evaluate the clinical potential of these lncRNAs in prognostic assessment of colorectal cancer. At the mechanistic level, the molecular mechanisms through which AC010319.3 regulates NK cell function and the immune microenvironment—particularly its specific downstream targets and signaling networks—remain unclear. Therefore, future studies will prioritize expanding the scope of external validation and further investigating the targets and signaling networks of AC010319.3 to facilitate its translation into clinical precision immunotherapy.

## 4 Conclusion

For the research, we integrated single-cell and multi-omics data to construct a prognostic model for CRC built on 16 NK cell-related lncRNAs, which operates independently of traditional parameters. We focused on exploring how AC010319.3 promotes tumor progression by inhibiting NK cell function, thereby providing potential targets and directions for precision immunotherapy in CRC.

## 5 Materials and methods

### 5.1 Single-cell transcriptomic data integration and NK cell population annotation

Single-cell transcriptomic data encompassing 10,468 cells from CRC were obtained from the GSE146771_Smartseq2 dataset in the GEO database. The data were analyzed using the TISCH2 platform (25). Dimensionality reduction was performed via Principal Component Analysis, and cell populations were identified and classified using the K-nearest neighbors (KNN) algorithm and the Louvain algorithm. Cell types were noted according to cell type-specific marker genes. Subsequently, the Wilcoxon rank-sum test was employed to identify genes with substantially differential expression in NK cell populations compared to all other cell populations. The selection criteria were an absolute fold change (|fold change|) ≥1.5 and a false discovery rate (FDR) <0.05 (26).

### 5.2 Cell-cell communication network analysis

We employed the CellChat tool (version 1.0.0) on the TISCH2 platform, based on the mass action model with a default interaction score threshold of 0.01, to analyze the expression models of detected L-R pairs across distinct cell populations and evaluate intercellular interactions. Using CellChat’s netVisual_circle, we mapped and visualized significant L-R interactions across cell subtypes. For each cell population, significant L-R pairs were identified and annotated as either “source” or “target” cells, with a statistical significance threshold of *P* < 0.05.

### 5.3 Functional enrichment of multicellular clusters

To gain deeper insights into the gene enrichment characteristics of distinct cell type populations, we performed GSEA on the TISCH2, ranking genes based on their logarithmic fold changes derived from differential analysis. Through an integrated approach combining KEGG pathway analysis, GO enrichment analysis, and GSEA, we identified and visualized substantially enriched biological pathways across each cell cluster (FDR = 0.05). This comprehensive methodology provided a robust foundation for elucidating functional enrichment patterns among heterogeneous cell populations.

### 5.4 TCGA data integration and NK cell-related lncRNA screening

We first retrieved gene expression profiles, clinical data, and somatic mutation information from 566 tumor samples and 44 normal colorectal tissue samples in TCGA database (27). Differential expression analysis was conducted to compare mRNA expression levels between tumor and non-tumor tissues. Building on this, we integrated the previously identified NK cell-associated differentially expressed genes and applied a correlation coefficient threshold of 0.4, thereby screening 3,837 NK cell-related lncRNAs. Finally, utilizing the “R.limma” gene expression analysis package, we identified 1,133 NK cell-related lncRNAs that showed substantial different manifestation between tumor and non-tumor tissues.

### 5.5 Construction of the NK-lncRNA prognostic model

CRC patients in this study were randomly divided into two cohorts at a 1:1 ratio (28), designated as the training and validation sets. Univariate COX regression analysis identified 42 NK cell-related lncRNAs associated with CRC prognosis in the training set. Subsequently, a multivariable Cox regression analysis was performed to set up a prognostic prediction model for CRC in the training set. Based on the coefficients assigned to each NK cell-related lncRNA by the model, Risk Score = 1.66835172119723 × LINC01354 expression + 0.382958718659199 × LINC02257 expression + 1.05788009854083 × AC010319.3 expression + 0.954271934790829 × AC009133.3 expression − 2.0577555829965 × THOC7-AS1 expression − 0.915211633569471 × LINC02100 expression − 1.06232138758457 × AL390719.3 expression + 0.876450507692965 × PLS3-AS1 expression + 0.66491751956799 × AC145423.2 expression + 0.689453742016689 × ALMS1-IT1 expression + 3.25571273528702 × ZFHX2-AS1 expression + 0.489382404521168 × AP003555.1 expression − 1.04327479032394 × AC103739.1 expression + 1.06508271262886 × NSMCE1-DT expression − 2.316681672941 × AL596214.1 expression − 3.18945858556773 × AC244100.2 expression. Patients were stratified into high- and low-risk groups based on this risk score. Kaplan-Meier analysis demonstrated significant survival differences between the risk groups. The model’s performance was evaluated using receiver operating characteristic (ROC) curves and further validated in both the validation dataset and the entire cohort. In addition, we have included an extra 76 independent clinical samples as an external validation dataset to further verify the reliability of the model.

### 5.6 Molecular characterization of high- and low-risk groups

We first employed the limma package in R to identify DEGs between risk groups using predefined thresholds (|log2(FC)| > 1, [FDR] < 0.05). The outcomes were projected via volcano plots and heatmaps to illustrate the distribution and magnitude of DEGs. Subsequently, GO enrichment analysis, encompassing biological processes, cellular components, and molecular functions, was performed using the clusterProfiler package in R. KEGG pathway enrichment analysis was also conducted, with significantly enriched pathways displayed as bubble plots. Furthermore, GSEA was used to assess biological function disparities between risk groups (29–31). (NES > 1, FDR < 0.05).

### 5.7 Immune microenvironment quantification

To investigate immune heterogeneity between risk groups, we integrated multiple bioinformatics approaches. Differential expression analysis of immune checkpoint-related genes was performed using the limma package in R. Data integration and reshaping were facilitated by the reshape2 package to ensure compatibility with downstream analyses. Immune-related functional states were evaluated via single-sample GSEA implemented in the GSVA package, enabling comprehensive quantification of immune activity. This multi-tool framework provided robust insights into the immune landscape, supporting translational research and clinical applications (32).

### 5.8 Consensus clustering-based molecular subtyping framework for CRC

Unsupervised consensus clustering was performed using the ConsensusClusterPlus package in R to delineate molecular subtypes of CRC (33). The optimal cluster number was decided by systematically evaluating three algorithmic outputs: CDF plots: Stability of clustering solutions was assessed by comparing slope changes in cumulative distribution curves across candidate cluster numbers. Consensus matrices (CM): These numerical matrices quantified the frequency with which sample pairs were assigned to the same cluster across iterative subsampling. Consensus heatmaps: Visual representations of consensus matrices highlighted clustering patterns, facilitating intuitive interpretation. The integration of these metrics established the optimal molecular classification, forming a theoretical foundation for prognostic model development.

### 5.9 Clinical sample collection

Pathological specimens from 76 CRC patients were obtained from Tongji Hospital, Tongji Medical College, Huazhong University of Science and Technology, China. Informed consent was provided by all patients, and the diagnosis of CRC was confirmed by two pathologists. This study was conducted in accordance with the ethical principles of the Declaration of Helsinki regarding ethical considerations and patient safety (Approval No.TJ-IRB20230934).

### 5.10 Flow cytometry cell sorting

Fresh tumor tissues were obtained from surgical resections of CRC patients. Fresh tissue samples were rinsed with pre-chilled PBS to remove blood and debris, then minced into 1–2 mm³ fragments. The tissue fragments were digested in an enzyme solution containing collagenase and DNase at 37°C in a constant-temperature shaker for 30–60 minutes, with gentle pipetting every 15 minutes. After digestion, the mixture was filtered through a 70 μm cell strainer. The filtrate was centrifuged at 1500 rpm for 5 minutes, and the supernatant was discarded to obtain a single-cell suspension. Cells were counted using trypan blue dye to exclude dead cells and assess suspension viability. The cell concentration was adjusted to 1×10^6^–5×10^6^ cells/ml. Subsequently, 100 μl of the cell suspension was transferred to a flow cytometry tube, and fluorescently labeled antibodies against CD45, CD3, and CD56 were added (at concentrations recommended by the manufacturer). The mixture was gently mixed and incubated at 4°C in the dark for 30 minutes. After incubation, 2 ml of pre-chilled PBS was added, followed by centrifugation at 1500 rpm for 5 minutes. The supernatant was discarded, and the washing step was repeated twice. Finally, cells were resuspended in 300–500 μl of pre-chilled PBS to prepare samples for analysis. Flow cytometry was used to sort tumor-infiltrating NK cells from fresh CRC tumor tissues. Single-cell suspensions were prepared from tumor tissues, and NK cells were identified and isolated as CD56+CD3− cells. The purity of CD56+CD3− NK cells in all samples exceeded >95%. Immediately after sorting, a portion of the NK cells (approximately 5 × 10^4^ to 1 × 10^5^ cells per sample) was used for RNA extraction and subsequent qPCR analysis of lncRNA expression levels, while the remaining cells were cryopreserved in liquid nitrogen for future experiments. qPCR detection of lncRNA expression in NK cells and CRC tumor tissues was performed using samples from 10 independent CRC patients. Cell sorting was conducted on a CytoFLEX SRT flow cytometer. Controls included FMO controls, with approximately 1 × 10^5^ events acquired per sample. During flow cytometry, appropriate gates were set to acquire 10^4^–10^5^ cells, and data were analyzed using FlowJo software. The gating strategy included: (1) FSC-H/SSC-H for lymphocytes, (2) FSC-H/FSC-A for singlets, (3) LDPB450/FSC-A to exclude dead cells, (4) CD45FITC-H/FSC-A to identify and isolate leukocytes, (5) CD3− to exclude T cells, and CD56+ to select NK cells (see **Supplementary Figure S10A** for details).

### 5.11 Cell culture

Human CRC cell lines (HCT-116, HT-29, SW480) were purchased from the Cell Bank of the Chinese Academy of Sciences (Shanghai, China) and cultured in Dulbecco’s Modified Eagle Medium (DMEM; EallBio, Beijing, China) supplemented with 10% fetal bovine serum (FBS; Gibco, California, USA) and 1% penicillin–streptomycin–amphotericin solution (NCM Biotech, Suzhou, China).

The human NK cell line NK92 was obtained from the Cell Bank of the Chinese Academy of Sciences (Shanghai, China). The cells were cultured in RPMI1640 medium (GIBCO) supplemented with 10% fetal bovine serum (FBS, GIBCO), 4 mM L-glutamine (GIBCO), 100 U/mL penicillin and 100 μg/mL streptomycin (Sigma-Aldrich), 10 mM HEPES (Sigma-Aldrich), and 100 U/mL recombinant human interleukin-2 (IL-2, Novartis).

### 5.12 Real-time quantitative PCR

Total RNA was extracted using the Trizol method (T9108,Takara, Dalian, China), and reverse transcription was performed using an enzyme kit. Subsequently, qRT-PCR was conducted using2 × ChamQ Universal SYBR qPCR Master Mix (Q711-02, Vazyme, Nanjing, China). The primer sequences used are listed in **Supplementary Table S1**.

### 5.13 Transfection

The pcDNA3.1 plasmid for AC010319.3 overexpression and the siRNA plasmid for AC010319.3 knockdown were obtained from Qingke Biological Company in Wuhan, China. Cells were seeded into 6-well plates at the correct density. Transfection was performed using lip­ofectamine 3000 following the provided instructions after 24 h.

### 5.14 Flow cytometry analysis

The NK92 cell line was used, and adherent cells were collected via trypsin digestion to prepare a single-cell suspension. To detect induced IFN-γ expression, cells were stimulated in complete medium containing PMA (50 ng/mL), ionomycin (1 μg/mL), and a protein transport inhibitor (e.g., monensin) at 37°C with 5% CO_2_ for 4–6 hours; all cells were uniformly subjected to stimulation conditions. After stimulation, cells were washed with pre-chilled PBS (centrifuged at 1500 rpm for 5 minutes, repeated twice). Then, 100 μl of cell suspension (adjusted to 1×10^6^–5×10^6^ cells/ml, with viable cell count confirmed by trypan blue staining) was transferred to a flow cytometry tube. Fluorescently labeled anti-CD56 antibody was added (at the manufacturer-recommended concentration), gently mixed, and incubated at 4°C in the dark for 30 minutes. After incubation, 2 ml of pre-chilled PBS was added, followed by centrifugation at 1500 rpm for 5 minutes; the supernatant was discarded, and the wash was repeated twice. Cells then underwent fixation and permeabilization (fixed with 4% paraformaldehyde for 20 minutes, followed by permeabilization with permeabilization buffer for 15–20 minutes). Subsequently, fluorescently labeled anti-IFN-γ and anti-GZMB antibodies were added simultaneously (at manufacturer-recommended concentrations) and incubated at 4°C in the dark for 30 minutes. After incubation, 2 ml of pre-chilled PBS was added, cells were centrifuged at 1500 rpm for 5 minutes, the supernatant was discarded, and the wash was repeated twice. Finally, cells were resuspended in 300–500 μl of pre-chilled PBS to prepare samples for acquisition. Flow cytometry analysis was performed on a CytoFLEX SRT flow cytometer, with FMO controls included. Appropriate channels were set during acquisition to collect 10^4^–10^5^ events per sample, and data were analyzed using FlowJo software. The gating strategy included: (1) FSC-H/SSC-H for lymphocytes, (2) FSC-H/FSC-A for singlets, and (3) CD56+ for NK cell identification (see **Supplementary Figure S10G** for details).

### 5.15 Co-culture assay

NK92 cells transfected with Vector and AC010319.3-OE were co-cultured with HCT116 cells (E:T = 1:1) for 48 hours. The cell suspension obtained after digestion was used for functional assays. To maintain cell viability, the mixed medium (RPMI-1640:DMEM = 1:1) containing 200 U/mL IL-2 was gently replaced every 12 hours without disturbing the adherent cells. Functional assays were repeated using NK92 cells transfected with si-NC and si-AC010319.3 co-cultured with HCT116.

### 5.16 Transwell invasion assay

After co-culture treatment, HCT116 cells were seeded into the upper chamber of a Matrigel-coated Transwell insert and resuspended in serum-free medium. The lower chambers were filled with medium supplemented with serum. After incubation, non-invaded cells on the upper surface of the membrane were removed, and invaded cells on the lower surface were fixed and stained. Finally, invaded cells were visualized under a microscope and quantified.

### 5.17 Cell proliferation assay

Cell proliferation was assessed using the Cell Counting Kit-8 (CCK-8; NCM Biotech). For the colony formation assay, 500 cells were seeded and incubated for two weeks. Colonies were fixed with paraformaldehyde for 0.5 hours, stained with crystal violet for 1 hour, counted, and photographed.

### 5.18 Analysis of statistics

All studies were conducted in R 4.4.3 and GraphPad Prism 8.0. Key methods included: Kruskal-Wallis tests for comparing ICGs expression, immune scores, and drug sensitivity across risk groups. Kaplan-Meier survival curves with log-rank tests (via the survival package) to assess survival disparities. Multivariable Cox proportional hazards models to evaluate joint effects of covariates. All tests were two-tailed, with statistical significance defined as *P* < 0.05. Significance levels were annotated as: *P < 0.001, P < 0.01, P < 0.05.

## Availability of data and materials

The primary data supporting this study were obtained from the Gene Expression Omnibus (GEO) database via the TISCH2 platform (http://tisch.comp-genomics.org/home/; PMID: 32302573), and additional data are available from the corresponding author upon reasonable request.

## Data Availability

The original contributions presented in the study are included in the article/**Supplementary Material**. Further inquiries can be directed to the corresponding authors.

## References

[1] Bray F Laversanne M Sung H Ferlay J Siegel RL Soerjomataram I . Global cancer statistics 2022: GLOBOCAN estimates of incidence and mortality worldwide for 36 cancers in 185 countries. CA. (2024) 74:229–63. doi: 10.3322/caac.21834, PMID: 38572751

[2] Bray F Ferlay J Soerjomataram I Siegel RL Torre LA Jemal A . Global cancer statistics 2018: GLOBOCAN estimates of incidence and mortality worldwide for 36 cancers in 185 countries. CA. (2018) 68:394–424. doi: 10.3322/caac.21492, PMID: 30207593

[3] Franzén AS Boulifa A Radecke C Stintzing S Raftery MJ Pecher G . Next-generation CEA-CAR-NK-92 cells against solid tumors: overcoming tumor microenvironment challenges in colorectal cancer. Cancers. (2024) 16. doi: 10.3390/cancers16020388, PMID: 38254876 PMC10814835

[4] Cai L Chen A Tang D . A new strategy for immunotherapy of microsatellite-stable (MSS)-type advanced colorectal cancer: Multi-pathway combination therapy with PD-1/PD-L1 inhibitors. Immunology. (2024) 173:209–26. doi: 10.1111/imm.13785, PMID: 38517066

[5] Westcott PMK Sacks NJ Schenkel JM Ely ZA Smith O Hauck H . Low neoantigen expression and poor T-cell priming underlie early immune escape in colorectal cancer. Nat Cancer. (2021) 2:1071–85. doi: 10.1038/s43018-021-00247-z, PMID: 34738089 PMC8562866

[6] Russick J Torset C Sun D Marmier S Merle N Voilin E . Tumor stage-driven disruption of NK cell maturation in human and murine tumors. iScience. (2024) 27:111233. doi: 10.1016/j.isci.2024.111233, PMID: 39583926 PMC11585790

[7] Maalej KM Merhi M Inchakalody VP Mestiri S Alam M Maccalli C . CAR-cell therapy in the era of solid tumor treatment: current challenges and emerging therapeutic advances. Mol Cancer. (2023) 22:20. doi: 10.1186/s12943-023-01723-z, PMID: 36717905 PMC9885707

[8] Sterner RC Sterner RM . CAR-T cell therapy: current limitations and potential strategies. Blood Cancer J. (2021) 11:69. doi: 10.1038/s41408-021-00459-7, PMID: 33824268 PMC8024391

[9] Lanuza PM Vigueras A Olivan S Prats AC Costas S Llamazares G . Activated human primary NK cells efficiently kill colorectal cancer cells in 3D spheroid cultures irrespectively of the level of PD-L1 expression. Oncoimmunology. (2018) 7:e1395123. doi: 10.1080/2162402x.2017.1395123, PMID: 29632716 PMC5889279

[10] Statello L Guo CJ Chen LL Huarte M . Author Correction: Gene regulation by long non-coding RNAs and its biological functions. Nat Rev Mol Cell Biol. (2021) 22:159. doi: 10.1038/s41580-021-00330-4, PMID: 33420484 PMC8095262

[11] Mattick JS Amaral PP Carninci P Carpenter S Chang HY Chen LL . Long non-coding RNAs: definitions, functions, challenges and recommendations. Nat Rev Mol Cell Biol. (2023) 24:430–47. doi: 10.1038/s41580-022-00566-8, PMID: 36596869 PMC10213152

[12] Li LY Zi H Deng T Li BH Guo XP Ming DJ . Autophagy-related long non-coding RNAs act as prognostic biomarkers and associate with tumor microenvironment in prostate cancer. Am J Cancer Res. (2024) 14:545–61. doi: 10.62347/xtdz5687, PMID: 38455413 PMC10915326

[13] Kang Z Yang J . Construction and validation of an autophagy-related long non-coding RNA signature to predict the prognosis of kidney renal papillary cell carcinoma. J Invest Med. (2022) 70:1536–44. doi: 10.1136/jim-2022-002379, PMID: 35725019

[14] Xu K Dai C Yang J Xu J Xia C Li J . Disulfidptosis-related lncRNA signatures assess immune microenvironment and drug sensitivity in hepatocellular carcinoma. Comput Biol Med. (2024) 169:107930. doi: 10.1016/j.compbiomed.2024.107930, PMID: 38199215

[15] Hui X Xue M Ren Y Chen Y Chen X Farooq MA . regulates the function of NK cells through the Gαs/CSK/ZAP70/NF-κB signaling pathway as a potential immune checkpoint. Sci Adv. (2025) 11:eadr9395. doi: 10.1126/sciadv.adr9395, PMID: 40043109 PMC11881902

[16] Xu K Xia P Gongye X Zhang X Ma S Chen Z . A novel lncRNA RP11-386G11.10 reprograms lipid metabolism to promote hepatocellular carcinoma progression. Mol Metab. (2022) 63:101540. doi: 10.1016/j.molmet.2022.101540, PMID: 35798238 PMC9287641

[17] Zhou L Jiang J Huang Z Jin P Peng L Luo M . Hypoxia-induced lncRNA STEAP3-AS1 activates Wnt/β-catenin signaling to promote colorectal cancer progression by preventing m(6)A-mediated degradation of STEAP3 mRNA. Mol Cancer. (2022) 21:168. doi: 10.1186/s12943-022-01638-1, PMID: 35986274 PMC9392287

[18] Tang M Song K Xie D Yuan X Wang Y Li Z . PSAT1 promotes the progression of colorectal cancer by regulating Hippo-YAP/TAZ-ID1 axis via AMOT. Mol Cell Biochem. (2024) 480(6):3647–68. doi: 10.1007/s11010-024-05194-8, PMID: 39739271 PMC12095340

[19] Zanconato F Battilana G Forcato M Filippi L Azzolin L Manfrin A . Transcriptional addiction in cancer cells is mediated by YAP/TAZ through BRD4. Nat Med. (2018) 24:1599–610. doi: 10.1038/s41591-018-0158-8, PMID: 30224758 PMC6181206

[20] Hosseini A Gharibi T Marofi F Babaloo Z Baradaran B . CTLA-4: From mechanism to autoimmune therapy. Int Immunopharmacol. (2020) 80:106221. doi: 10.1016/j.intimp.2020.106221, PMID: 32007707

[21] Mortezaee K . HHLA2 immune-regulatory roles in cancer. Biomed Pharmacother Biomed Pharmacother. (2023) 162:114639. doi: 10.1016/j.biopha.2023.114639, PMID: 37011487

[22] Wu Z Hu Z Han X Li Z Zhu Q Wang Y . The BET-Bromodomain Inhibitor JQ1 synergized ABT-263 against colorectal cancer cells through suppressing c-Myc-induced miR-1271-5p expression. Biomed Pharmacother Biomed Pharmacother. (2017) 95:1574–9. doi: 10.1016/j.biopha.2017.09.087, PMID: 28950657

[23] Agarwal E Chaudhuri A Leiphrakpam PD Haferbier KL Brattain MG Chowdhury S . Akt inhibitor MK-2206 promotes anti-tumor activity and cell death by modulation of AIF and Ezrin in colorectal cancer. BMC Cancer. (2014) 14:145. doi: 10.1186/1471-2407-14-145, PMID: 24581231 PMC3941258

[24] Grasso S Pereira GJS Palmeira-Dos-Santos C Calgarotto AK Martínez-Lacaci I Ferragut JA . Autophagy regulates Selumetinib (AZD6244) induced-apoptosis in colorectal cancer cells. Eur J Med Chem. (2016) 122:611–8. doi: 10.1016/j.ejmech.2016.06.043, PMID: 27448918

[25] Han Y Wang Y Dong X Sun D Liu Z Yue J . TISCH2: expanded datasets and new tools for single-cell transcriptome analyses of the tumor microenvironment. Nucleic Acids Res. (2023) 51:D1425–d31. doi: 10.1093/nar/gkac959, PMID: 36321662 PMC9825603

[26] Xu KQ Gong Z Yang JL Xia CQ Zhao JY Chen X . B-cell-specific signatures reveal novel immunophenotyping and therapeutic targets for hepatocellular carcinoma. World J Gastroenterol. (2024) 30:3894–925. doi: 10.3748/wjg.v30.i34.3894, PMID: 39350784 PMC11438648

[27] Xu K Xia P Liu P Zhang X . A six lipid metabolism related gene signature for predicting the prognosis of hepatocellular carcinoma. Sci Rep. (2022) 12:20781. doi: 10.1038/s41598-022-25356-2, PMID: 36456877 PMC9715694

[28] Li J Xia C Li Y Liu H Gong C Liang D . Effects of NK cell-related lncRNA on the immune microenvironment and molecular subtyping for pancreatic ductal adenocarcinoma. Front Immunol. (2024) 15:1514259. doi: 10.3389/fimmu.2024.1514259, PMID: 39872533 PMC11770056

[29] Kanehisa M Furumichi M Sato Y Matsuura Y Ishiguro-Watanabe M . KEGG: biological systems database as a model of the real world. Nucleic Acids Res. (2025) 53:D672–d7. doi: 10.1093/nar/gkae909, PMID: 39417505 PMC11701520

[30] Kanehisa M Goto S . KEGG: kyoto encyclopedia of genes and genomes. Nucleic Acids Res. (2000) 28:27–30. doi: 10.1093/nar/28.1.27, PMID: 10592173 PMC102409

[31] Kanehisa M . Toward understanding the origin and evolution of cellular organisms. Protein Sci. (2019) 28:1947–51. doi: 10.1002/pro.3715, PMID: 31441146 PMC6798127

[32] Hu G Yao H Wei Z Li L Yu Z Li J . A bioinformatics approach to identify a disulfidptosis-related gene signature for prognostic implication in colon adenocarcinoma. Sci Rep. (2023) 13:12403. doi: 10.1038/s41598-023-39563-y, PMID: 37524774 PMC10390519

[33] Wilkerson MD Hayes DN . ConsensusClusterPlus: a class discovery tool with confidence assessments and item tracking. Bioinf (Oxford England). (2010) 26:1572–3. doi: 10.1093/bioinformatics/btq170, PMID: 20427518 PMC2881355

