# Supplementary material for: NK cell-associated long non-coding RNAs reveal heterogeneity of colorectal cancer immune microenvironment
Source: Front Immunol. 2025 Nov 13;16:1615942. doi: 10.3389/fimmu.2025.1615942 (PMC12657463; doi:10.3389/fimmu.2025.1615942)
Supplement: Supplementary file 11 [file Table1.docx]

**Supplementary Information**

**Title**

**NK Cell-Associated Long Non-Coding RNAs Reveal Heterogeneity of Colorectal Cancer Immune Microenvironment**

| **Supplementary Table S1. The primer sequences used for qRT-PCR** | | | |
| --- | --- | --- | --- |
| Gene | | Sequence (5’-3’) | |
| LINC01354 | F: CTGTTGTTTTCCTCGCGGTG | |  |
|  | R: AAGCTCCAGACGTGTTCTCG | |  |
| LINC02257 | F: CTCAAGTGTGTCATGGGCCT | |  |
|  | R: TTTTGGCGGGGATTCTACCC | |  |
| AC010319.3 | F: AAGCTCGGTGCTTAACGGAA | |  |
|  | R: GCTGCGTATCCCACAGATGA | |  |
| AC009133.3 | F: TGAGGTGAGGCTTGGGTAAC | |  |
|  | R: GGACTCCAGAGCCTGATGAC | |  |
| THOC7-AS1 | F: GCAGGAAAAGGCAACCTGTG | |  |
|  | R: AGGAGCTAAGTTTCCTCGCC | |  |
| LINC02100 | F: CCCATCCGGGATTTCTCCAC | |  |
|  | R: TGTGGGAAGTTTACATTGTTTGGT | |  |
| AL390719.3 | F: AACTGTGCTGGTTGCTTCCT | |  |
|  | R: GACCCTGCCATAGCACAACT | |  |
| PLS3-AS1 | F: AGGTAGGAAGCCTGGGGTAG | |  |
|  | R: AGCATCCTTTGTTGGGAGGG | |  |
| AC145423.2 | F: AACTGTGCTGGTTGCTTCCT | |  |
|  | R: GACCCTGCCATAGCACAACT | |  |
| ALMS1-IT1 | F:TCCAGATCCCTCAGCAAGCA | |  |
|  | R:TGCCCAATTATGCTGTGACCA | |  |
| ZFHX2-AS1 | F: AGAGGGTAGAGTGCCTCCTG | |  |
|  | R: ATTCGACCCCCTGAAAACCC | |  |
| AP003555.1 | F: GCAGCCCTCGATGACCAATA | |  |
|  | R: GGAAAAGGGCAGGTGCTTTG | |  |
| AC103739.1 | F: AGATAGGGTCCCCTGAACACA | |  |
|  | R: CGGAGGTGCAGCTTGAACTA | |  |
| NSMCE1-DT | F: GGGAAGAAGAAGTGCGACGA | |  |
|  | R: ACCTCTGTGAGCATTGGGTG | |  |
| AL596214.1 | F: CCCACACCTCTTGCTCCAAA | |  |
|  | R: TCGTGGTGGAAGGCAAAAGT | |  |
| AC244100.2 | F: ATGTGTTGTTGGTGGCCTGA | |  |
|  | R: TGCAGGTTCTGCTTCGAGTT | |  |

GAPDH F:GGAGCGAGATCCCTCCAAAAT

R:GGCTGTTGTCATACTTCTCATGG
