# Supplementary material for: NK cell-associated long non-coding RNAs reveal heterogeneity of colorectal cancer immune microenvironment
Source: Front Immunol. 2025 Nov 13;16:1615942. doi: 10.3389/fimmu.2025.1615942 (PMC12657463; doi:10.3389/fimmu.2025.1615942)
Supplement: Supplementary file 12 [file Table2.docx]

**Supplementary Figure 1**

Heatmap showing the differential expression of core transcription factors in different cell types of CRC.

**Supplementary Figure 2**

(A, B) Dot plot showing transcription factors significantly expressed in NK cells of CRC.

**Supplementary Figure 3**

Heatmap visually depicting enriched KEGG pathways upregulated by different cell subpopulations.

**Supplementary Figure 4**

Heatmap visually depicting enriched KEGG pathways downregulated by different cell subpopulations.

**Supplementary Figure 5**

(A) Heatmap visually depicting enriched GOBP pathways upregulated in different cell subpopulations.
(B) Heatmap visually depicting enriched GOBP pathways downregulated in different cell subpopulations.

**Supplementary Figure 6**

(A) Heatmap visually depicting enriched GOCC pathways upregulated in different cell subpopulations.
(B) Heatmap visually depicting enriched GOCC pathways downregulated in different cell subpopulations.

**Supplementary Figure 7**

(A) Heatmap visually depicting enriched GOMF pathways upregulated in different cell subpopulations.
(B) Heatmap visually depicting enriched GOMF pathways downregulated in different cell subpopulations.

**Supplementary Figure 8**

(A) Heatmap showing upregulated immune gene sets in different cell subpopulations.
(B) Heatmap showing downregulated immune gene sets in different cell subpopulations.

**Supplementary Figure 9**

Box plot showing drug sensitivity to different molecular types of drugs.

**Supplementary Figure 10**

(A) Isolation of NK cells from colorectal cancer patient tissues by flow cytometry.

(B) Comparison of lncRNA expression between NK cell lines and colorectal cancer cell lines.

(C) Survival analysis from the TCGA database showing the impact of relevant lncRNA expression levels on prognosis.

(D) Impact of relevant lncRNA expression levels on prognosis in clinical samples (n=76).

(E) Establishment of an NK cell model overexpressing AC010319.3.

(F) Establishment of an AC010319.3-knockdown NK cell model

(G) NK Cell Gating Strategy and FMO Controls
